# Transfusion Reactions in Pediatric Patients: An Analysis of 5 Years of Hemovigilance Data From a National Center for Children's Health in China

**DOI:** 10.3389/fped.2021.660297

**Published:** 2021-05-28

**Authors:** Kai Guo, Xiaohuan Wang, Huimin Zhang, Mengjian Wang, Shanshan Song, Shuxuan Ma

**Affiliations:** Department of Transfusion Medicine, Beijing Children's Hospital, Capital Medical University, National Center for Children's Health, Beijing, China

**Keywords:** blood transfusion, pediatric patient, children, transfusion reaction, hemovigilance system

## Abstract

**Objective:** This study aimed to describe transfusion reactions of pediatric patients from a National Center for Children's Health in China and to examine reaction incidents, reaction types by blood transfusion, and the associated blood products resulting in transfusion reactions.

**Methods:** We compared transfusion reaction rates, among platelets, plasma, and red blood cells (RBCs) using a retrospective analysis of pediatric patients treated with blood transfusion based on data from the National Center for Children's Health (Beijing, China) by a hemovigilance reporting system from January 2015 to December 2019.

**Results:** Over the past 5 years, 165 reactions were reported, and the overall incidence was 1.35‰ (95% CI: 1.14–1.55‰; 165/122,652); for each separate year, the incidences were 1.25‰ (95% CI: 0.76–1.74‰; 25/20,035; 2015), 1.09‰ (95% CI: 0.65–1.52‰; 24/22,084; 2016), 1.66‰ (95% CI: 1.14–2.18‰; 39/23,483; 2017), 1.36‰ (95% CI: 0.92–1.81‰; 36/26,440; 2018) and 1.34‰ (95% CI: 0.93–1.75‰; 41/30,610; 2019). Transfusion reaction incidents by person included 0.37‰ (95% CI: 0.21–0.53‰; 21/56,815) RBCs, 2.98‰ (95% CI: 2.33–3.64‰; 79/26,496) platelets and 1.65‰ (95% CI: 1.25–2.05‰; 65/39,341) frozen plasma. According to the analysis by blood products, the incidence of transfusion was 0.34‰ (95% CI: 0.20–0.48‰; 23/66,958) for RBCs, 3.21‰ (95% CI: 2.50–3.92‰; 78/24,318.5) for platelets, and 0.94‰ (95% CI: 0.71–1.17‰; 64/67,912) for frozen plasma. Transfusion reactions were most commonly associated with platelets, followed by plasma and RBC transfusions. The types of blood transfusion reactions were mainly allergic reactions (86.67%) and febrile non-hemolytic transfusion reactions (FNHTRs, 4.24%). The disease types of pediatric patients with transfusion reactions were concentrated among those with blood system diseases. A total of 80.61% of children with transfusion reactions had a previous blood transfusion history.

**Conclusions:** Transfusion reactions are still relatively common in pediatric patients, and additional studies are necessary to address the differences in reaction rates, especially allergic and FNHTRs. Robust hemovigilance systems do include a special section dedicated to children will further the understanding of these reactions and trends, and prospective randomized clinical controlled trials may need to be conducted to perform preventive and corrective measures.

## Introduction

A century ago, pediatric medicine was defined based on its specialty. However, there remains a deficit of age-appropriate evidence base (1). Children physiology and pathology differ significantly as compared to adults during growth and development (2, 3), some indications may be in common with adults, but others are unique to the physiology of infancy or disease processes found only in childhood. Consequently, clinical guidelines used for adult patients are not fully adapted to pediatric patients in terms of product types, modifications, doses, transfusion indications, blood product selections, potential transfusion reactions. However, currently, most of pediatric guidelines and consensus guidance (such as pediatric transfusion) are not completely based on studies and clinical practice of children (4, 5).

According to a 2-year cohort study from Peltoniemi et al., there was a significant reduction in mortality when children were treated in pediatric units rather than in adult and child mixed wards (6). In a word, it is necessary to conduct more studies to understand the unique characteristics of the pediatric patient population.

Transfusions are the therapeutic procedures performed in infants and children in many clinical conditions, and are also associated with substantial risks (7), including serious transfusion reactions [~1% (8)], such as hemolytic transfusion reactions (9), transfusion-associated circulatory overload TACO (10) and transfusion-related acute lung injury TRALI (11). Understanding the transfusion reactions in pediatric patients for the use of blood products is essential both to pediatricians wishing to optimize transfusion practice and to blood providers needing to plan the provision of blood products for children. However, data describing outcomes and, in particular, transfusion reactions among pediatric patients are lacking (12–15). A retrospective analysis was conducted of 9 children's hospitals and 35 adult hospitals in the United States from Vossoughi et al.'s study (16), which included pediatric and adult patients who had a reported reaction to blood transfusion of any blood component. Compared to adults, pediatric patients had double the rate of transfusion reactions (16). The difference in transfusion reaction rates between children and adults will raise the question of whether blood transfusion of pediatric patients requires a special consideration. However, there are limited reported data on transfusion reactions in Chinese pediatric patient population.

The hemovigilance reporting system is a tool to improve blood transfusion quality, especially blood transfusion safety (17). Data gathering in a hemovigilance reporting system plays a key role in monitoring how changes in transfusion practice affect the incidence and severity of transfusion reactions and ensures patient safety with blood transfusions (18). The national Chinese Hemovigilance Network (CHN) was established and became a member of the International Hemovigilance Network in 2017. The first annual Chinese hemovigilance report was published in 2021 (19). However, the harmonization of data is likely still evolving, particularly in transfusion reactions of pediatric patients.

In this study, we used a hemovigilance reporting system in our hospital to evaluate the incidence, type, and associated blood products resulting in transfusion reactions in children. The aims of our study were to expand the existing knowledge on pediatric patients' responses to blood transfusion based on a hemovigilance reporting system in China and to provide data comparing the relative incidence of various transfusion reactions to all blood products in pediatric patients to better inform clinical doctors of the relative likelihood of reactions with respect to both blood products and patient ages.

## Methods

### Study Design and Patient Selection

This was a retrospective observational study. The study population was comprised of patients from a National Center for Children's Health of China (Beijing), which is one of the largest general children's hospitals in China. Our hemovigilance reporting system is integrated into the blood transfusion information management system as a general strategy of risk management at the National Center for Children's Health, Beijing, China. There is mandatory reporting of transfusion reactions in the hospital by clinical doctors, and an electronic database was used to store these data.

We performed a retrospective transfusion reaction analysis using information extracted from our hemovigilance reporting system from January 2015 to December 2019. All patients had to meet the following inclusion criteria: age 0 days to 18 years and transfusion reactions following blood transfusions. All of the information was reported with the following personal identifying information masked before reporting: identity document, patient name, birth date, history of blood transfusion, etc.

### Transfusion Reaction Case Classification Criteria

Transfusion reactions are defined based on the CHN report (19): allergic transfusion reaction, FNHTR, acute hemolytic transfusion reaction (AHTR), hypotensive transfusion reaction (HTR), TACO, TRALI, transfusion-associated dyspnea (TAD), delayed hemolytic transfusion reaction (DHTR), delayed serologic transfusion reaction (DSTR), transfusion-associated graft vs. host disease (TA-GVHD), posttransfusion purpura (PTP), transfusion-transmitted infection (TTI) and unknown. The criteria were applied from the United State CDC recommendations (http://www.cdc.gov/nhsn/PDFs/Biovigilance/BV-HV-protocol-current.pdf) for each reaction.

### Blood Product Unit

One red blood cell (RBC) unit was processed from 200 ml whole blood with an additive solution (usually CPDA-1), leading to a final volume of 160 ± 10% ml, a hematocrit of 50–65% and Hb ≥ 20 g (RBC in additive solution) or 150 ± 10% ml, a hematocrit of 45–60%, Hb ≥ 18 g and leukocytes ≤ 2.5 × 10^6^ (RBC in additive solution leukocytes reduced); the latter RBC units were prestored leukoreduced. These two types of blood components are usually prepared as RBC products in our blood bank. Plasma was prepared from whole blood and frozen at −20°C or colder within 6 to 8 h of collection. On average, one unit contained 100 ml from 200 ml whole blood. The plasma was not pathogen-reduced. The concentrated platelet units were manufactured from units of whole blood that had not been cooled < 20°C. On average, 10 donor units were calculated one unit of platelet treatment, which was equal to one unit of apheresis platelets (250–300 ml, containing ≥ 2.5 × 10^11^ platelets). The concentrated platelets and the apheresis platelets were not leukoreduced and then stored between 20 and 24°C for up to 5 days. The apheresis platelets are usually prepared the platelet products in our blood bank. ABO-compatible platelets are usually provided in our medical center.

### Statistical Analysis

Continuous variables are displayed as the median and interquartile ranges (IQRs; min, max). Categorical variables are described as frequencies (percentages).

## Results

### Characteristics of Pediatric Patients With Transfusion Reactions

A total of 165 pediatric patients between January 2015 and December 2019 met the inclusion criteria. Among all patients, 21 (12.73%) received at least 0.5 units of RBCs, 78 (47.27%) received at least 0.5 units of plasma and 66 (40.00%) received at least 0.5 units of platelets treatment during their hospitalization. The types of blood transfusion reactions were mainly allergic reactions (urticarial/rash, including 141 cases; anaphylactic shock, including 2 cases), FNHTRs (including seven cases), and unknown reactions (including 15 cases), which main symptoms included tachycardia (including 12 cases), chills (including 10 cases), chest tightness (including six cases) and body shaking (including one case). These diseases of pediatric patients with transfusion reactions were concentrated among children with blood system diseases, including acute lymphoblastic leukemia (including 73 cases), non-Hodgkin's lymphoma (including nine cases), aplastic anemia (including seven cases), hemophagocytic syndrome (including two cases), pancytopenia (including 12 cases) and others, such as Henoch-Schonlein purpura (including nine cases). A total of 80.61% of children with transfusion reactions had a previous history of blood transfusion. The cohort characteristics of pediatric patients with transfusion reactions and the distribution of the types as well as the frequency of each reported incident over the past 5 years of our study are summarized in Table 1.

**Table 1:** Baseline information of pediatric patients with transfusion reactions.

**Characteristics**	**Values *n* (%)**
Cases of patients included	165
Age, years	3 (6, 9; 0.50, 14.17)
Gender	
Boys	118 (71.52)
Girls	47 (28.48)
Blood products	
RBC	21 (12.73)
plasma	78 (47.27)
Platelet	66 (40.00)
Blood groups	
A	34 (20.61)
AB	12 (7.27)
B	66 (40.00)
O	53 (32.12)
Reaction types	
Allergic reaction	143 (86.67)
FNHTR	7 (4.24)
Unknown^*^	15 (9.09)
Disease types	
Blood system diseases	103 (62.42)
Others	62 (37.58)
Blood transfusion history	133 (80.61)

* *Unknown reaction types include coughing, chest tightness, wheezing, abdominal discomfort, chills, tachycardia, body shaking and cyanosis of the lips*.

### Transfusion Reaction Incidence Reported From 2015 to 2019

**Figure 1:**
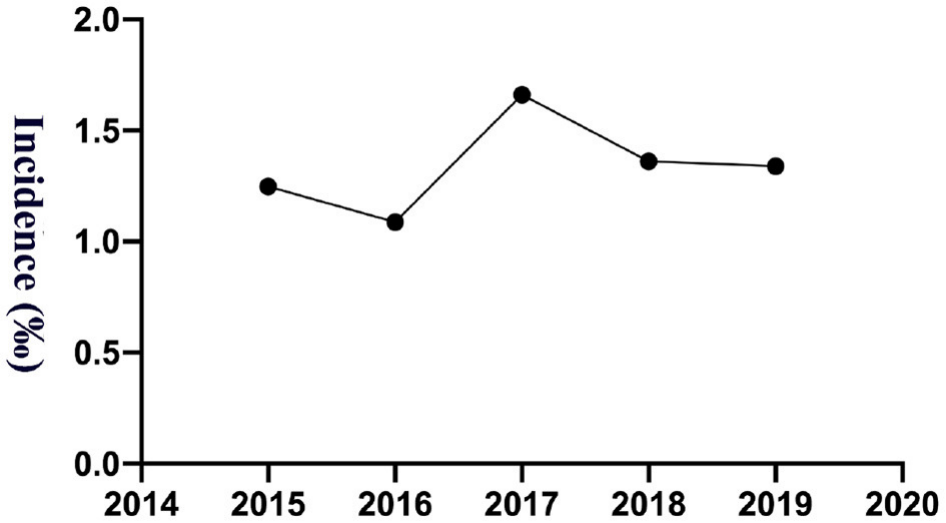
The incidence of transfusion reactions from 2015 to 2019.

**Figure 2:**
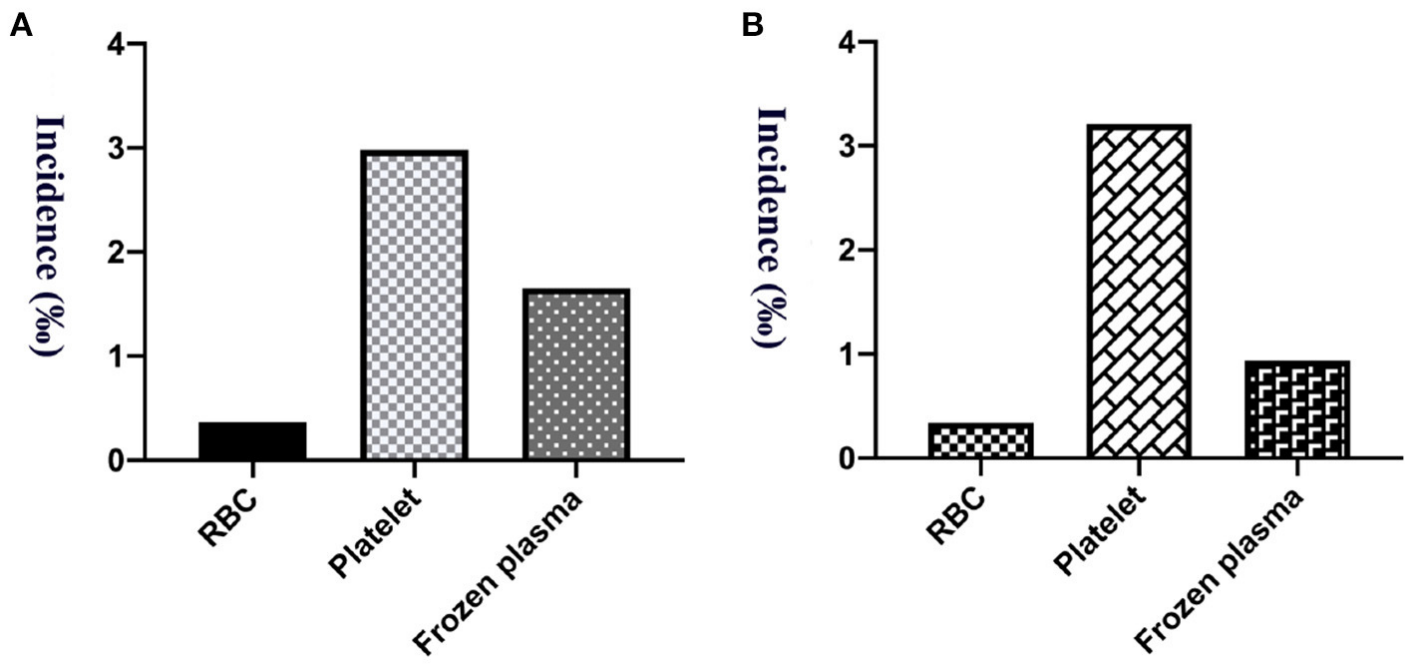
The incidence of transfusion reaction from 5 years of RBCs, platelets and frozen plasma. **(A)** Per person count. **(B)** Per blood product count.

The study calculated the transfusion reaction incidence from 2015 to 2019, and the overall incidence was 1.35‰ (95% CI: 1.14–1.55‰; 165/122,652), among which per year it was 1.25‰ (95% CI: 0.76–1.74‰; 25/20,035; 2015), 1.09‰ (95% CI: 0.65–1.52‰; 24/22,084; 2016), 1.66‰ (95% CI: 1.14–2.18‰; 39/23,483; 2017), 1.36‰ (95% CI: 0.92–1.81‰; 36/26,440; 2018) and 1.34‰ (95% CI: 0.93–1.75‰; 41/30,610; 2019), respectively, as shown in Figure 1. Transfusion reaction incidents by person included 0.37‰ (95% CI: 0.21–0.53‰; 21/56,815) RBCs, 2.98‰ (95% CI: 2.33–3.64‰; 79/26,496) platelets and 1.65‰ (95% CI: 1.25–2.05‰; 65/39,341) frozen plasma as shown in Figure 2A.

A total of 159,188.5 labile blood products were distributed among our hospitalized and outpatient children from 5 years, among which 66,958 were RBC units, 24,318.5 units of platelet treatment and 67,912 units of frozen plasma. The incidence of transfusion reactions was 0.34‰ (95% CI: 0.20–0.48‰; 23/66,958) for RBCs, 3.21‰ (95% CI: 2.50–3.92‰; 78/24,318.5) for platelets, and 0.94‰ (95% CI: 0.71–1.17‰; 64/67,912) for frozen plasma, as shown in Figure 2B. Transfusion reactions were most commonly associated with platelets, followed by plasma and RBC transfusions.

## Discussion

Blood transfusions are lifesaving therapies and one of the most common procedures performed in hospitals; however, they can result in transfusion reactions, which can be infectious (20) or more commonly non-infectious (21). Although clinicians and transfusion specialists have taken many blood safety measures, transfusion reactions remain an important cause of transfusion patients of the associated high morbidity and mortality (7, 22). Transfusion reactions are defined as adverse reactions, which are undesirable responses or effects in patients temporally associated with the infusion of blood products (23). In general, transfusion reactions include allergic reactions, FNHTR, TRALI, TACO, transfusion-associated dyspnea, hypotensive transfusion reaction, bacteremia, transfusion-related sepsis, delayed serologic transfusion reaction, posttransfusion purpura, TA-GVHD and acute or delayed hemolysis, and isolated hypotension (7). These transfusion reactions can be asymptomatic, mild or potentially fatal. Accurate estimates of transfusion reaction rates, analyses the types of transfusion reactions are essential for effective diagnosis, treatment and prevention.

Hemovigilance is the process of surveillance and alarm during the blood transfusion pathway (24). However, the approaches to hemovigilance are somewhat different between countries, which explains why the results are dissimilar among these different systems, such as India (25), Poland (26), etc. In China, the approaches to hemovigilance are even somewhat different among cities. Actually, it is a legal obligation to report adverse effects of blood transfusions. Our report demonstrated a pediatric hemovigilance system in a National Center for Children's Health in China by analyzing the transfusion reactions reported over a period of 5 years.

Reactions related to infusion of blood products were reported by the clinicians using a standard preformatted form, giving a synopsis of the reaction based on the hemovigilance system. From 2015 to 2019, the transfusion reaction incidence was relatively stable (1.09–1.66‰), still relatively common, but lower than Vossoughi's report (5.38‰) (16) and Oakley FD's study (6.16‰) (15) in the United State, De Pascale MR's report (2.63‰) in Italy (27), Gauvin F's report for pediatric patients from intensive care units (General pediatrics, Hemato-Oncology, General surgery, Cardiac surgery, Cardiology Gastrohepatology, Orthopedics and Others) (15.94‰) in Canada (28), Pedrosa AK's report for oncology, general pediatric patients and intensive care units (14.39‰) in Brazil (29), Hu W's report (8.18‰) in China (30). It is possible that the age of the pediatric population was differently calculated within age < 21 (15), 8 to 19 years (27) or 0 to 285.8 months (28) in some reports. In addition, the reported rates of transfusion reactions suffer from high heterogeneity, likely the result of differences in definitions, observational vigilance during blood transfusions, and reporting practice, as well as pathophysiological differences between pediatric patient populations, even among different races. Regardless, the low rate of transfusion reactions means that many studies are under powered for accurate comparisons of products.

In this study, the types of blood transfusion reactions were mainly allergic reactions and FNHTRs. This was not entirely surprising and as expected, the present results were consistent with the data reported by other researchers (15, 16, 23, 28, 31). A single-center investigation reported a significantly higher incidence of transfusion reactions among pediatric patients with FNHTRs, hypotensive transfusion reactions, and allergic transfusion reactions as the main contributors (15). Within the pediatric population, there was an increased incidence of allergic transfusion reactions and FNHTRs compared to the adult population (15). Statistically higher rates of allergic reactions and FNHTRs were previously observed in pediatric patients (16, 31). According to the International Hemovigilance Network (INH) database, information was provided on 132.8 million blood components issued in the period 2006 to 2012; among the severe adverse reactions, the most common were allergic reactions (33.4%) and FNHTRs (32.3%) (23). Moreover, their transfusion reaction incidence had the similar sex ratio to our report, and reactions were more common in male patients (15). The leucocytes or the mediators from leucocytes may be underlying cause of these two major types of transfusion reactions (allergic reactions and FNHTRs) in the pediatric patient population (31).

According to our reports, platelet units gave rise to more transfusion reactions than frozen plasma and RBCs. Overall, transfusion reactions were most commonly associated with platelets, followed by plasma and RBCs. Kracalik et al. have demonstrated that transfusion reaction incidents were higher among apheresis and pathogen-reduced platelets than apheresis RBCs by quantified transfusion reaction risks based on the data during 2013 to 2018 from the National Healthcare Safety Network Hemovigilance Module in United State, and allergic reactions and FNHTRs were also most common (32). According to Gonzalez DO's report, platelet transfusions were associated with higher risks for postoperative complications in pediatric patients for solid tumors along with volume dependent (33). Saadah et al. (34) evaluated 7 years of annual aggregate hemovigilance data from 23 countries on plasma transfusion reactions. Plasma transfusions can result in relatively high rates of transfusion reaction incidents. Moreover, the most commonly reported plasma transfusion reactions are allergic reactions (IQR: 5.6–72.2 reactions/10^5^ units transfused), FNHTRs (0–9.1), TRALI, and TACO (34). A total of 80.61 % of children with transfusion reactions had a previous history of blood transfusion. The diseases of pediatric patients with transfusion reactions were concentrated among those with blood system diseases in our center. These results are basically consistent with the data reported by Yanagisawa et al. (31) and Hu et al. (30). Allergic transfusion reactions were significantly more frequent in children with hematological and malignant diseases and children who received higher number of blood transfusions (30, 31). A detailed analysis of some of the transfusion reaction incident reports is necessary. It can reveal complex deviations and/or failures of the procedures in place in the hospital, allowing for rapid advice and implementation of corrective and preventive measures, to improve blood transfusion safety.

In summary, blood transfusions are important supportive care in pediatric patients, but transfusion reactions are also the problem, long-term monitoring of blood transfused patients are essential. Our data provide insight into pediatric transfusion reactions. Future studies are necessary to address the differences in transfusion reaction rates, especially allergic reactions and FNHTRs, and to further address blood transfusion practices in unique and vulnerable populations. That is to say, additional studies are necessary to clarify transfusion reactions in the pediatric patient population. Simultaneously, the national hemovigilance system is important and needs to choose among several strategies for blood product needs based in part on comparative transfusion safety. It is also important that improve knowledge level of blood transfusion safety among pediatricians (35, 36) for quickly recognize transfusion reaction, stop the blood transfusion, evaluate and support the patient.

There are several limitations need to be mentioned in this study. First, the national CHN was founded in 2017 (19), so harmonization of data is likely still evolving. Therefore, our study also only provided a single-institution retrospective observational report on pediatric transfusion reactions. Second, blood products that were irradiated or leukoreduced were not well-characterized. Third, the cohort of transfusion reactions was characterized but no information was provided about the baseline patient population receiving transfusions at the hospital. Fourth, there may be some blood transfusion reactions that have not been recognized and reported by pediatricians. Recognizing accurately and reporting transfusion reactions to the Blood Bank or Department of Transfusion Medicine in the hospital are important part of ensuring blood transfusion safety and supporting hemovigilance efforts. Fifth, there was no grading of the severity of the transfusion reactions.

## Data Availability Statement

The raw data supporting the conclusions of this article will be made available by the authors, without undue reservation.

## Ethics Statement

The studies involving human participants were reviewed and approved by The Ethics Committee of Beijing Children's Hospital, Capital Medical University. Written informed consent from the participants' legal guardian/next of kin was not required to participate in this study in accordance with the national legislation and the institutional requirements.

## Author Contributions

KG wrote the drafts of the manuscript, performed the statistical analyses, and reviewed and revised the manuscript. KG, XW, HZ, MW, SS, and SM contributed to the acquisition of data. SM coordinated and supervised data collection and entry and critically reviewed the manuscript. All of the authors contributed to the article and approved the submitted version and agreed to be accountable for the results published.

## Conflict of Interest

The authors declare that the research was conducted in the absence of any commercial or financial relationships that could be construed as a potential conflict of interest.
